# Restriction of V3 region sequence divergence in the HIV-1 envelope gene during antiretroviral treatment in a cohort of recent seroconverters

**DOI:** 10.1186/1742-4690-10-8

**Published:** 2013-01-18

**Authors:** Astrid Gall, Steve Kaye, Stéphane Hué, David Bonsall, Richard Rance, Gregory J Baillie, Sarah J Fidler, Jonathan N Weber, Myra O McClure, Paul Kellam

**Affiliations:** 1 Wellcome Trust Sanger Institute, Wellcome Trust Genome Campus, Hinxton, Cambridge CB10 1SA, UK; 2 Jefferiss Research laboratories, Faculty of Medicine, Imperial College London, St Mary’s Campus, Norfolk Place, London, W2 1PG, UK; 3 UCL/MRC Centre for Medical Molecular Virology, Division of Infection and Immunity, University College London, 46 Cleveland St, London, W1T 4JF, UK

**Keywords:** HIV, Envelope, Deep sequencing, Primary infection, Diversity, Divergence, HAART, Short course antiretroviral therapy, AIDS, Coreceptor tropism

## Abstract

**Background:**

Dynamic changes in Human Immunodeficiency Virus 1 (HIV-1) sequence diversity and divergence are associated with immune control during primary infection and progression to AIDS. Consensus sequencing or single genome amplification sequencing of the HIV-1 envelope *(env)* gene, in particular the variable (V) regions, is used as a marker for HIV-1 genome diversity, but population diversity is only minimally, or semi-quantitatively sampled using these methods.

**Results:**

Here we use second generation deep sequencing to determine inter-and intra-patient sequence heterogeneity and to quantify minor variants in a cohort of individuals either receiving or not receiving antiretroviral treatment following seroconversion; the SPARTAC trial. We show, through a cross-sectional study of sequence diversity of the *env* V3 in 30 antiretroviral-naive patients during primary infection that considerable population structure diversity exists, with some individuals exhibiting highly constrained plasma virus diversity. Diversity was independent of clinical markers (viral load, time from seroconversion, CD4 cell count) of infection. Serial sampling over 60 weeks of non-treated individuals that define three initially different diversity profiles showed that complex patterns of continuing HIV-1 sequence diversification and divergence could be readily detected. Evidence for minor sequence turnover, emergence of new variants and re-emergence of archived variants could be inferred from this analysis. Analysis of viral divergence over the same time period in patients who received short (12 weeks, ART12) or long course antiretroviral therapy (48 weeks, ART48) and a non-treated control group revealed that ART48 successfully suppressed viral divergence while ART12 did not have a significant effect.

**Conclusions:**

Deep sequencing is a sensitive and reliable method for investigating the diversity of the *env* V3 as an important component of HIV-1 genome diversity. Detailed insights into the complex early intra-patient dynamics of *env* V3 diversity and divergence were explored in antiretroviral-naïve recent seroconverters. Long course antiretroviral therapy, initiated soon after seroconversion and administered for 48 weeks, restricts HIV-1 divergence significantly. The effect of ART12 and ART48 on clinical markers of HIV infection and progression is currently investigated in the SPARTAC trial.

## Background

Primary infection by Human Immunodeficiency Virus type 1 (HIV-1) is usually initiated by a single or small number of infecting virions [1]. Quantitative assessment of HIV-1 evolution can be determined by measuring HIV sequence diversity, the genetic variation at a given time, and divergence, the genetic distance to a reference point, usually the founder virus. In the absence of immune pressure HIV-1 diversifies and diverges from the founder virus by random mutation. This occurs during virus dissemination from the site of primary infection and the establishment of a peak viremia [1]. Extensive viral diversity arises from the high error rate of the HIV-1 reverse transcriptase, the rapid virus turnover, and the large population size. Diversity is constrained upon initiation of the CD8 cytotoxic T lymphocyte (CTL) response [2]. Under intense CTL selection, escape variants occur and expand within the infectious virus pool. Single genome amplification (SGA) sequencing and deep amplicon sequencing by Roche/454 technology show many viral CTL escape variants can exist in parallel at the time of declining viral load and establishment of viral set point in both HIV and Simian Immunodeficiency virus (SIV) infections [3,4].

Longitudinal analysis of HIV-1 sequence variation following seroconversion shows virus divergence and diversity accumulate co-linearly at approximately 1% per year [5]. Sequence diversity increases, eventually plateaus, and then in some cases declines over time. During the diversity plateau, however, divergence continues to increase until, at a later time, divergence stabilisation is also reached [5]. The emergence of CXCR4-tropic variants may occur immediately prior to the peak of diversity, with stabilisation of divergence occurring during periods of low CD4+ T cell numbers [5]. Although the dynamics of HIV-1 diversity during primary infection are well studied, less is known about the dynamics of diversity and divergence following seroconversion and clinical latency, or the effect of antiretroviral therapy (ART) on altering the course of diversification and divergence. High HIV-1 envelope (*env)* diversity is linked to slower disease progression and effective immune control. Specifically, Heteroduplex Tracking Assay (HTA) analysis of populations of HIV-1 *env* V1/V2 and V4/V5 regions showed that high levels of sequence diversity were linked with slower disease progression and that rapid CD4+ T cell decline was associated with lower *env* diversification [6]. Using the same assay over shorter time intervals revealed that selective pressures on V1/V2 and V4/V5 are intense and continually evolving [7]. Together this suggests that continued virus replication, in the absence of drug therapy, but in the presence of adaptive immune responses drives divergence and diversity.

That intense selective pressure leads to HIV-1 mutational escape has also been shown in the development of antiviral drug resistance. Interestingly, recent data as revealed by deep sequencing suggest that rather than providing a strong selective pressure on the *env* gene and constraining diversity, treatment with the CCR5 antagonist Vicriviroc resulted in V3 *env* diversity increasing during treatment and was coupled with an expansion of rare sequence variants during drug resistance selection [8]. This suggested that continued evolutionary pressure resulted in the observable exploration of the sequence space. Whether immune selective pressures result in the same drive of diversification has not been determined to the same detail by HTA which is a semi-quantitative and non sequence based method. In contrast, for patients on successful highly active antiretroviral therapy (HAART), an absence of evolution has been shown for both the *env* C2-V3-C3 [9] and the protease encoding region [10]. However, these studies were based on consensus and clonal sequencing, and did not analyse the impact of the length of therapy on the diversification and divergence of the virus.

Sequencing using second generation sequencing technology (Roche/454 Life Sciences) [11] has proven to be a promising alternative to traditional clonal sequencing. It surpasses the potential of clonal sequencing with regard to sensitivity, throughput, and ability to quantify minor variants. However, the drawbacks of a higher sequencing error rate [8,12-14] and the potential generation of recombinants during sample preparation by PCR [15,16] have to be taken into account when minor variants are analyzed. Deep sequencing of the *env* V3 loop has been utilized in a limited number of chronically infected patients to detect small minority X4-tropic viruses before [17] and during ART with a CCR5 antagonist [8], as well as their emergence after interruption of this therapy [18] or HAART [12].

Here, we show the application of deep sequencing of the HIV-1 *env* V3 to determine the diversity of HIV-1 in patients at primary infection with HIV-1 subtype B prior to initiating ART. We show the detailed and complex intra-patient dynamics of diversity and divergence following primary infection. Importantly we provide evidence that ART initiated soon after seroconversion can affect the dynamics of HIV diversification and divergence.

## Results

### Sequence data and filtering

Deep sequencing data for the *env* V3 region of 80 patient and 3 control samples were generated using 12 Genome Sequencer FLX Instrument runs. Either 1/4 or 1/8 region of a PicoTiterPlate was used for each pool of 5 to 12 samples. For one sample pool, sequencing yielded an average of 34.13 MB (megabases) of total data from 115284 reads with a read length of 297.87 ± 52.69 (mean ± SD). The average number of initial reads generated was 13929 (508–88144) for patient samples and 16954 (15739–19215) for control samples. Various filtering steps were applied to the deep sequencing data (see Methods and Additional file 1: Table S1). The resulting average sequencing depth for patient samples was 6908-fold (354–29387). For the control sequences from cloned HIV envelopes E6 and H8, the sequencing depth following data filtering was 9783-fold (E6) and 15524-fold (H8); and for the “mixed” HIV *env* control E6:H8, the sequencing depth was 11491-fold. Phred quality scores for non-consensus bases in the *env* V3 region were analysed for all samples (Additional file 2: Figure S1). The median Phred score was 32.50 with a range of 21.17 to 39.86. Therefore, the median of the probability of a nucleotide being called incorrectly was 0.05% (range 0.76%–0.01%). However, as Phred scores only account for base calling errors, we also assessed the overall error rate of our entire amplification and sequencing process.

### Error rates in 454 deep sequencing

**Figure 1:**
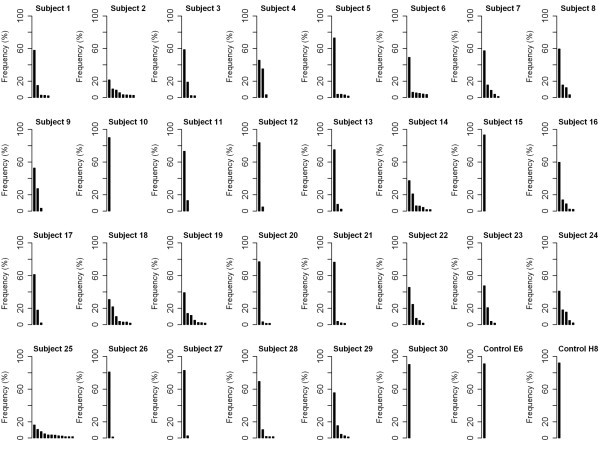
**Sequence variants of the *env *V3 in patients at primary HIV infection and control samples.** Major and minor sequence variants found in 30 subjects at primary infection with HIV-1 subtype B and two control samples, E6 and H8 (*in vitro* transcribed RNA from cloned and sequenced *env* genes), are shown. Each bar represents one unique sequence variant. Only variants with frequencies above the 1.5% cut-off are displayed.

*In vitro* transcribed RNA from two cloned and sequenced *env* genes (E6 and H8) as well as a mixture of both (E6:H8) with a molar ratio of 9:1 served as controls for total processing error rates. Reverse transcription, nested PCR, deep sequencing, assembly and data analysis were performed as for the patient samples. In a first step, process error rates were assessed using the controls E6 and H8. A total of 213 (E6) and 279 (H8) unique sequences that diverged by one or more nucleotides from the cloned *env* sequence were identified (Additional file 1: Table S1), therefore of the total sequences obtained 90.89% (E6) and 92.08% (H8) were identical to the respective plasmid sequence (Figure 1). The most frequent minor variant which was not identical to the plasmid sequence obtained a frequency of 1.17% (E6) and 0.55% (H8), respectively. For each of the 102 (E6) or 105 (H8) nucleotide positions of the *env* V3 loop of the alignment, we calculate that 99.89% (E6) and 99.90% (H8) of the nucleotides were correct, yielding the frequency of errors introduced in the total sequencing process as 0.11% and 0.10%, respectively (Table 1). Mismatches rather than gaps accounted for the majority of the errors within our data processing pipeline. The sequence accuracy in non-homopolymeric regions and homopolymeric regions, defined as repeats of three or more identical bases and the flanking non-identical bases [13], was compared. Errors were more frequent in homopolymeric regions consistent with the 454 technology, an average of 0.33% (E6) and 0.18% (H8) erroneous nucleotides were found here as opposed to 0.07% in non-homopolymeric regions for both clones.

**Table 1:** Determination of sequence accuracy of control samples

**Sequence accuracy**		**Control E6**			**Control H8**		
		**Mean**	**Minimum**	**Maximum**	**Mean**	**Minimum**	**Maximum**
**Overall**	**% correct nt**	99.89	98.37	100.00	99.90	98.96	100.00
	**% erroneous nt**	0.11	0.00	1.63	0.10	0.00	1.04
	**% mismatches**	0.09	0.00	1.61	0.07	0.00	0.48
	**% gaps**	0.02	0.00	0.94	0.03	0.00	0.70
**Non-homopolymeric regions**	**% correct nt**	99.93	99.72	100.00	99.93	99.57	100.00
	**% erroneous nt**	0.07	0.00	0.28	0.07	0.00	0.43
	**% mismatches**	0.06	0.00	0.27	0.05	0.00	0.38
	**% gaps**	0.01	0.00	0.07	0.02	0.00	0.39
**Homopolymeric regions**^**a**^	**% correct nt**	99.67	98.37	99.99	99.82	98.96	100.00
	**% erroneous nt**	0.33	0.01	1.63	0.18	0.00	1.04
	**% mismatches**	0.25	0.01	1.61	0.11	0.00	0.48
	**% gaps**	0.08	0.00	0.94	0.07	0.00	0.70

^a^ Repeats of 3 or more identical bases + flanking non-identical bases.

Ratios of major and minor variants and the ability of our methods to generate recombinant sequences were also assessed by using the “mixed” control E6:H8 combined at a ratio of 9 and 1. The two major sequence variants were identical to the plasmid sequences of E6 (77.90% of the sequences) and H8 (11.86%). All remaining sequences were present at low frequencies of ≤ 0.40% (not shown). At least one recombinant sequence was identified within these minor variants at a very low frequency of 0.02% (2/11491 reads). A second recombinant sequence with a frequency of 0.01% (1/11491 reads) differed from the first only by an inversion of two nucleotides at the end of a homopolymer tract (not shown). Our error profile was almost identical to that of Wang *et al*. [13], therefore using the same Poisson model of 454 sequencing errors, we estimated that we require a depth of greater than 750-fold to detect a sequence present at 1.5%, and at that level we were conservatively well in excess of both homopolymeric, non-homopolymeric, base calling errors and recombinant error rates.

### Sequence diversity of the HIV *env* V3 at primary infection

Deep sequencing of the *env* V3 region of 30 patients at primary infection with HIV-1 subtype B showed a complex pattern of major and minor sequence variants (Figure 1). The composition of major and minor variants differed between individuals with the major sequence variant accounting for up to 93.3% of the sequences (subject 15) or only 16.2% (subject 25). Between one (subjects 10, 15 and 30) and 12 (subject 25) error corrected genuine sequence variants (present at greater than 1.5% of the total sequences) were found in each individual. Of note, for the individuals with one major variant the sequences are effectively clonal. Although frequencies of major and minor sequence variants appear informative we wished to examine HIV-1 *env* sequence diversity in the more formal framework of Shannon Entropy.

### Coreceptor tropism of *env* V3 variants at primary infection

Utilization of the CCR5 or CXCR4 receptor as an HIV coreceptor was predicted based on *env* V3 variants of the 30 patients as well as two control samples. Results of predictions by (a) the PSSM method using the X4R5 matrix, (b) the PSSM method using the SINSI matrix and (c) the Geno2pheno [coreceptor] tool are shown in Additional file 3: Figure S2. For the majority of the variants, CCR5 usage was predicted using all methods. Sequence variants associated with virus using the CXCR4 coreceptor were clearly identified in subject 18. The 2^nd^ (frequency 21.82%), 5^th^ (3.29%) and 7^th^ (1.82%) most frequent variants were associated with CXCR4 coreceptor usage, totaling 26.93% of the sequences found in this patient. CXCR4 coreceptor predictions were consistent for the three methods for the variants with frequencies of 21.82% and 3.29%. For these two variants, PSSM scores applying the X4R5 matrix were −2.35 and PSSM scores using the SINSI matrix were 1.93; the false positive rate (FPR) for CXCR4 usage obtained by the Geno2pheno [coreceptor] tool was 1.7%. However, for the variant with a frequency of 1.82%, the PSSM SINSI matrix (score −0.45) and the Geno2pheno [coreceptor] tool (FPR 1.5%) predicted CXCR4 usage, while the PSSM X4R5 matrix predicted a dual-tropic phenotype (score −6.88).

### *Env* sequence diversity at primary infection is independent of clinical markers of HIV infection

**Figure 2:**
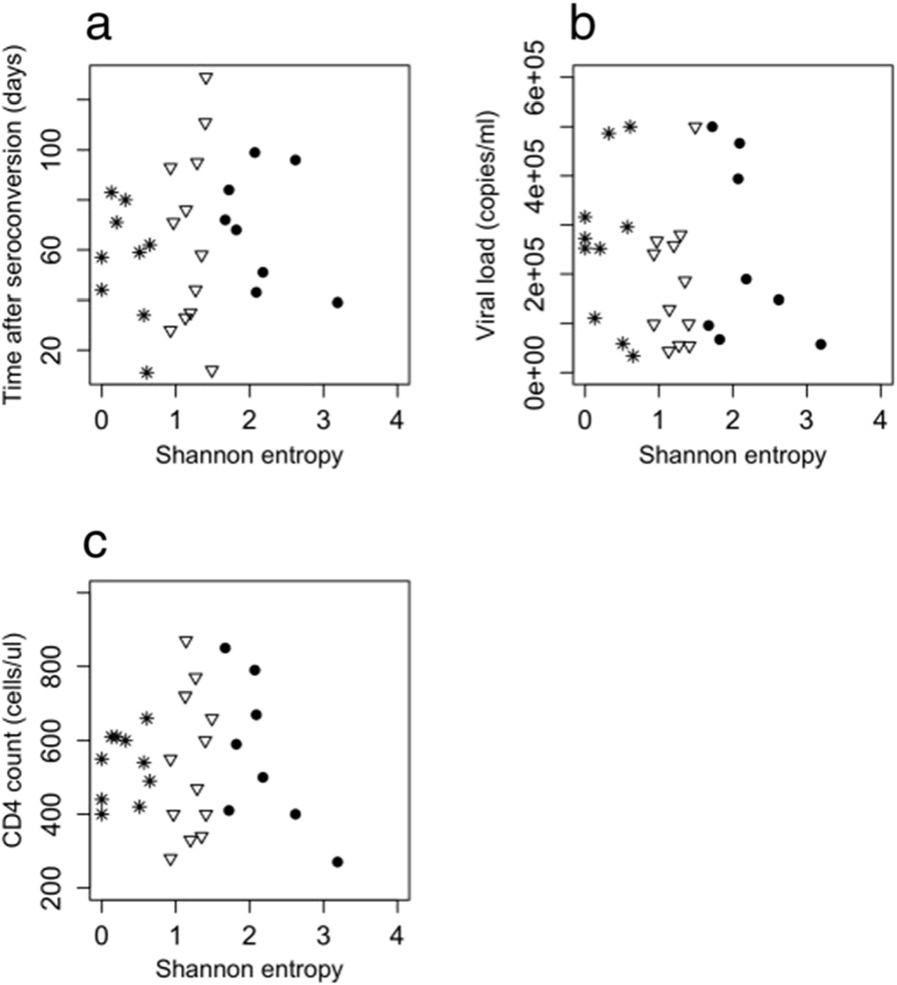
**Correlation of the Shannon Entropy with clinical markers of HIV infection.** The correlation with (**a**) the time after seroconversion, (**b**) the viral load, and (**c**) the CD4 cell count is shown for 30 subjects at primary infection with HIV. As the time to seroconversion is not known for subject 15, it is not included in (**a**). Patients were classified into three groups based on the sequence diversity. Group 1 is defined by a low sequence diversity (Shannon Entropy 0–0.75) and is shown as stars. Group 2 is defined by a medium sequence diversity (Shannon Entropy 0.75–1.5) and is shown as triangles. Group 3 is defined by a high sequence diversity (Shannon Entropy > 1.5) and is shown as dots.

The Shannon Entropy was used as a metric for population and sequence diversity, as it summarizes the number of sequence variants and their frequencies in a single value. Accordingly, a strong correlation (R^2^ = 0.874) of the Shannon Entropy with the number of sequence variants identified for each individual was observed (Additional file 4: Figure S3). In this inter-individual, cross sectional analysis, the Shannon Entropy did not correlate with the time from seroconversion (R^2^ = 0.011), viral load (R^2^ = 0.013) or CD4 cell count (R^2^ = 0.001) (Figure 2a, b, c). In addition, no correlation of these parameters with the sequence depth was observed (not shown, R^2^ between 0.001 and 0.149). Therefore, we arbitrarily assigned individuals into three groups based on the Shannon Entropy as a measure for population complexity. Group 1 was defined by low sequence diversity at primary infection (Shannon Entropy 0–0.75), group 2 by a medium sequence diversity (Shannon Entropy 0.75–1.5), and group 3 by a high sequence diversity (Shannon Entropy > 1.5) (Figure 2). From each of these groups, we selected one non-treated individual where serial samples were available to follow the change of diversity and divergence over time.

### Dynamics of sequence diversity of the *env* V3 following primary infection

**Figure 3:**
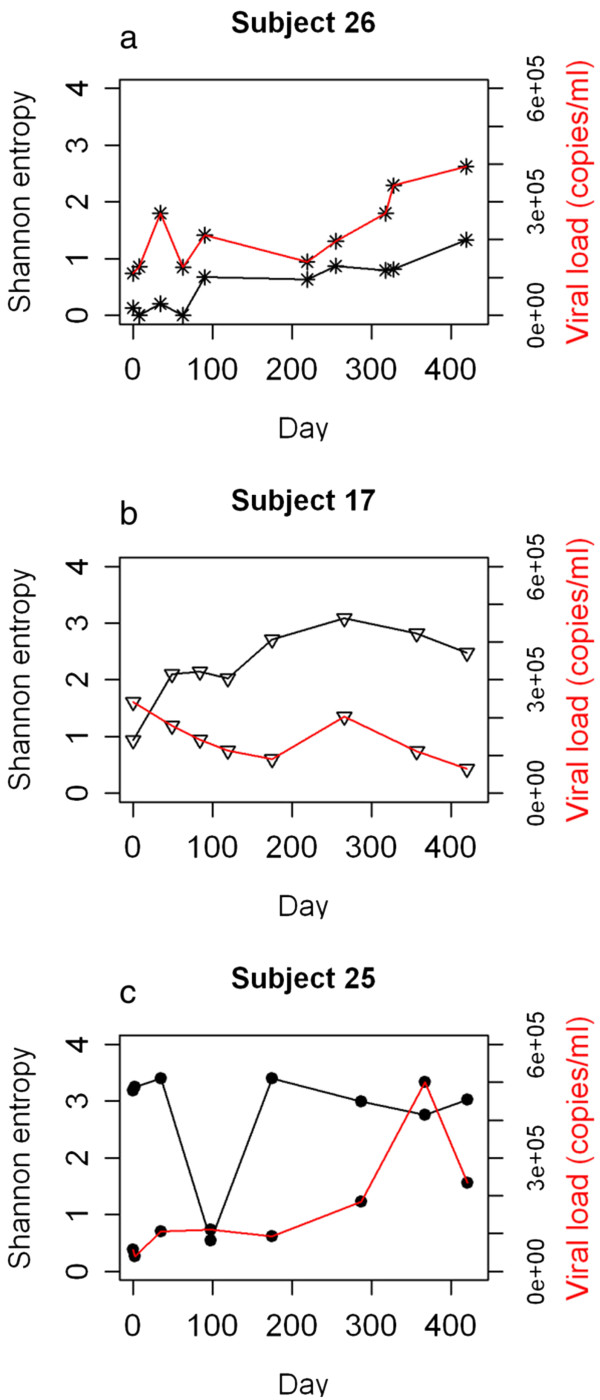
**Diversity and viral load over a time of ~ 60 weeks for three subjects.** The Shannon Entropy is used as a measure for sequence diversity. Results are shown for (**a**) subject 26, with low sequence diversity, (**b**) subject 17, with medium sequence diversity, and (**c**) subject 25, with high sequence diversity at primary HIV infection.

We sequenced serial samples over ~ 60 weeks following primary infection from three individuals of the non-treated control group and used the Shannon Entropy as a measure of sequence diversity. A general trend of increasing population diversity was observed for two individuals (Subject 26 and 17). Subject 26 had the lowest sequence diversity among all individuals at primary infection (Shannon Entropy 0.13), where more than one sequence variant was initially identified. Both the sequence diversity and the viral load in this subject fluctuated over time, with an overall trend of increasing viral load and sequence population diversity (Figure 3a). A trend towards a correlation of the Shannon Entropy with the viral load (not shown, R^2^ = 0.565) was observed. Subject 17 had ‘medium’ sequence diversity at primary infection. The sequence diversity after 60 weeks was considerably higher than at primary infection. In contrast, the viral load declined over time (Figure 3b). Both viral load and sequence diversity showed a transient peak at day 266, however, overall the Shannon Entropy did not correlate with viral load (not shown, R^2^ = 0.247). Subject 25 had the highest sequence diversity among all individuals following primary infection (Shannon Entropy 3.19). This degree of diversity was generally stable over time (Shannon Entropy 3.03 after ~ 60 weeks) except from a substantial decline in diversity at day 98 (Figure 3c). The viral load increased slowly over the time course but with a strong transient peak at day 367. The Shannon Entropy did not correlate with the viral load (not shown, R^2^ = 0.002).

### Dynamics of sequence divergence of the *env* V3 following primary infection

**Figure 4:**
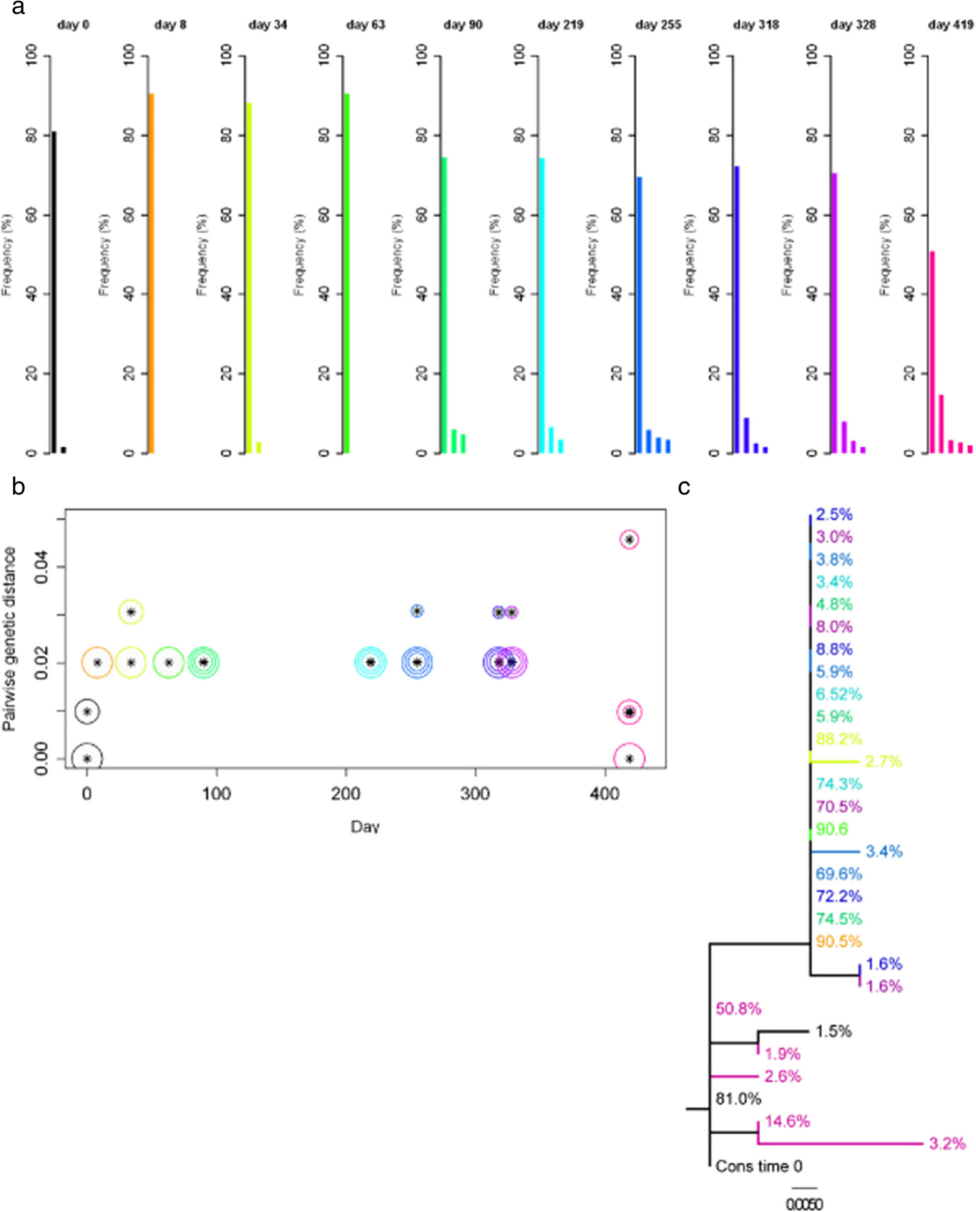
**Sequence variants of the *env *V3, divergence and Neighbor-joining tree for subject 26.** Samples over a time of ~ 60 weeks were analysed; with day 0 shown in black, and a rainbow colour scheme ranging from orange to pink applied to the remaining time points. (**a**) Sequence variants of the *env* V3. Each bar stands for one unique sequence variant. Only variants with frequencies above the 1.5% cut-off are displayed. (**b**) Divergence of *env* V3 sequence variants. Divergence is shown as the pairwise genetic distance (number of nucleotide substitutions per site) of a sequence variant at a given time from the consensus sequence at time 0. Genetic distances were estimated under the General Time Reversible model of nucleotide substitution, with proportion of invariable sites and gamma-distributed rate heterogeneity (GTR + I + G). Sizes of the circles around the symbols represent the frequencies of the sequence variants. The largest circle at each time point marks the major variant; the 2^nd^, 3^rd^ and 4^th^ most abundant variants are shown with gradually smaller circles; from the 5^th^ most abundant variant circles have the same size. Note that the scale of the y-axis in this figure is different from Figures 5 and 6. (**c**) Neighbor-joining tree of *env* V3 sequence variants. The tree was reconstructed under the GTR + I + G model of nucleotide substitution. Branches are labeled with frequencies of sequence variants as depicted in (a). The scale bar represents the number of nucleotide substitutions per site.

**Figure 5:**
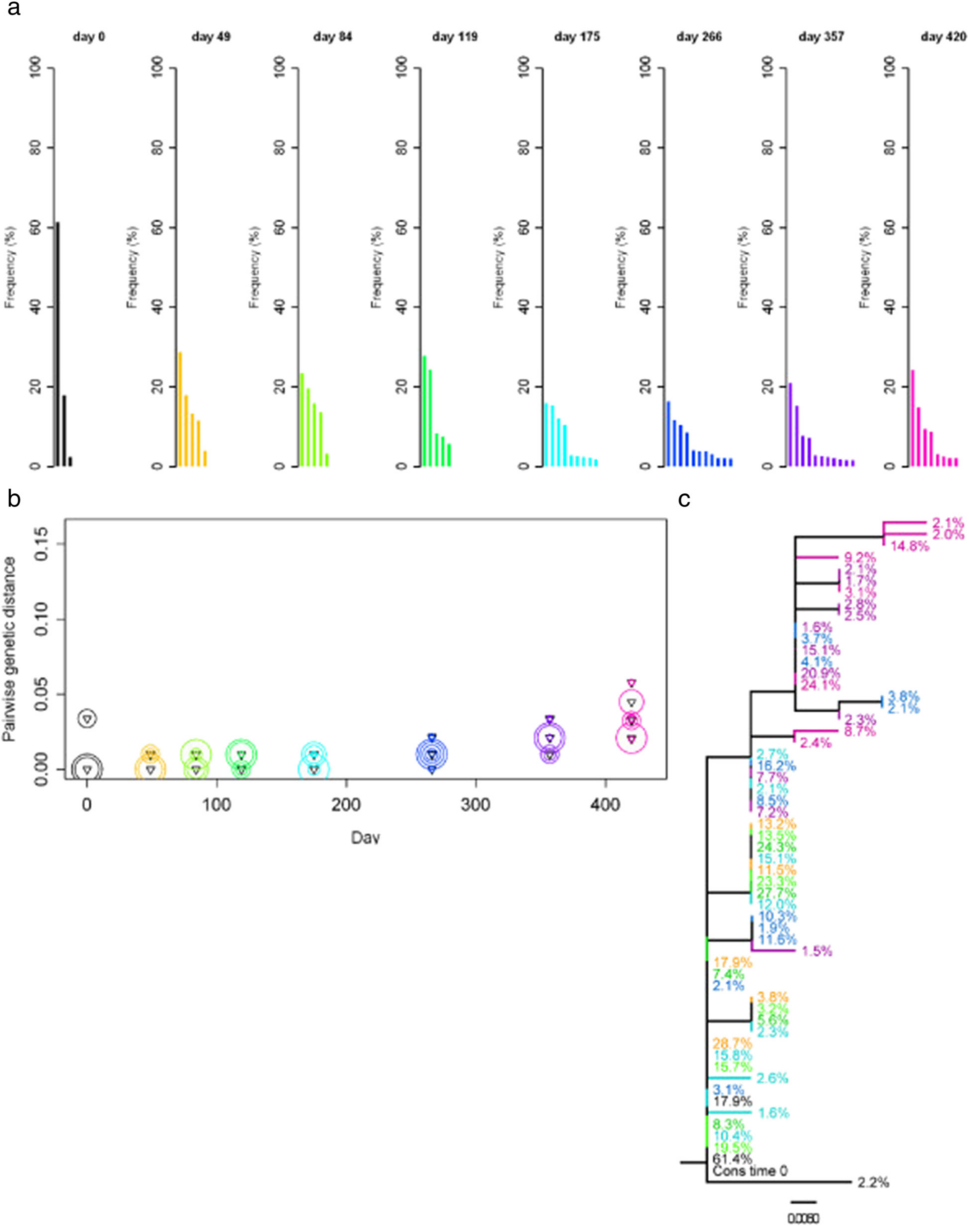
**Sequence variants of the *env *V3, divergence and Neighbor-joining tree for subject 17.** Samples over a time of ~ 60 weeks were analysed; with day 0 shown in black, and a rainbow colour scheme ranging from orange to pink applied to the remaining time points. The remaining details are as per Figure 4.

**Figure 6:**
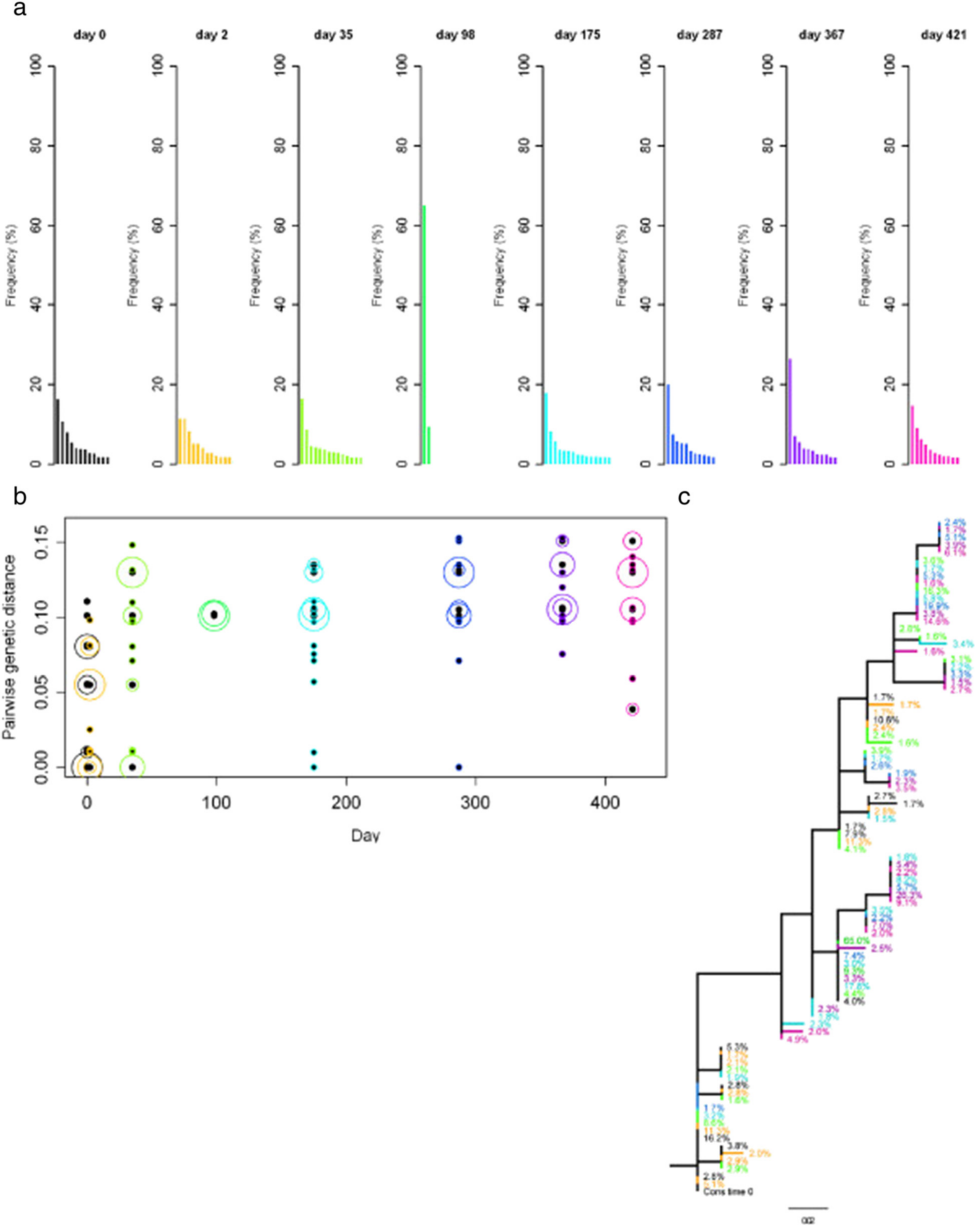
**Sequence variants of the *env *V3, divergence and Neighbor-joining tree for subject 25.** Samples over a time of ~ 60 weeks were analysed; with day 0 shown in black, and a rainbow colour scheme ranging from orange to pink applied to the remaining time points. The remaining details are as per Figure 4.

We investigated sequence divergence of the serial samples of subjects 26, 17 and 25 by determining the genetic distance of *env* V3 sequence variants at each time point relative to the consensus sequence at the first time point (Figures 4, 5 and 6). Pairwise genetic distances for each variant and Neighbor-joining trees were used to visualise the complex dynamics of deep sequence changes over time. Subject 26 had ‘low’ sequence diversity initially. After the early divergence until day 8, sequence diversity and divergence were then stable over 320 days (Figure 4). Interestingly, the major variant present at day 419 appeared to be a re-emergence of the dominant sequence variant detected immediately post seroconversion. In addition the minor sequence variants present at day 419 were also closely related, but distinct from the variants present at day 8. Subject 17 who had a ‘medium’ sequence diversity following primary infection showed a slow but continuously increasing divergence from the consensus virus over 420 days (Figure 5b) with the later time points showing the outgrowth of a distinct HIV population (Figure 5c). It is also clear that early, post seroconversion plasma virus variants are not present as detectable minority species at day 420 and that little sequence diversification occurred between days 49 to 266. Subject 25 had ‘high’ sequence diversity at primary infection, and sequence divergence continued over 421 days (Figure 6). Visualisation using Neighbor-joining trees coupled with frequency analysis revealed a complex biology in this individual. Two genetically distinct populations (I and II) were seen at early time points, both of which continued to diversify and diverge over 421 days. No evidence of recombinant sequences was observed. The frequencies of the two populations were similar up to day 35 and between day 175 and 421. Interestingly, a transient dominant variant with a frequency of 65% arose from population I at 98 days and whilst later variants in group I were related to this variant it did not lead to a complete population turnover and commensurate decrease in the frequency of group II variants at later times. After analysing the dynamics of sequence diversity and divergence in these three non-treated individuals in detail, we sought to explore the impact of ART on viral diversification and divergence over time in all 30 patients.

### Influence of ART on the divergence of the viral population

**Figure 7:**
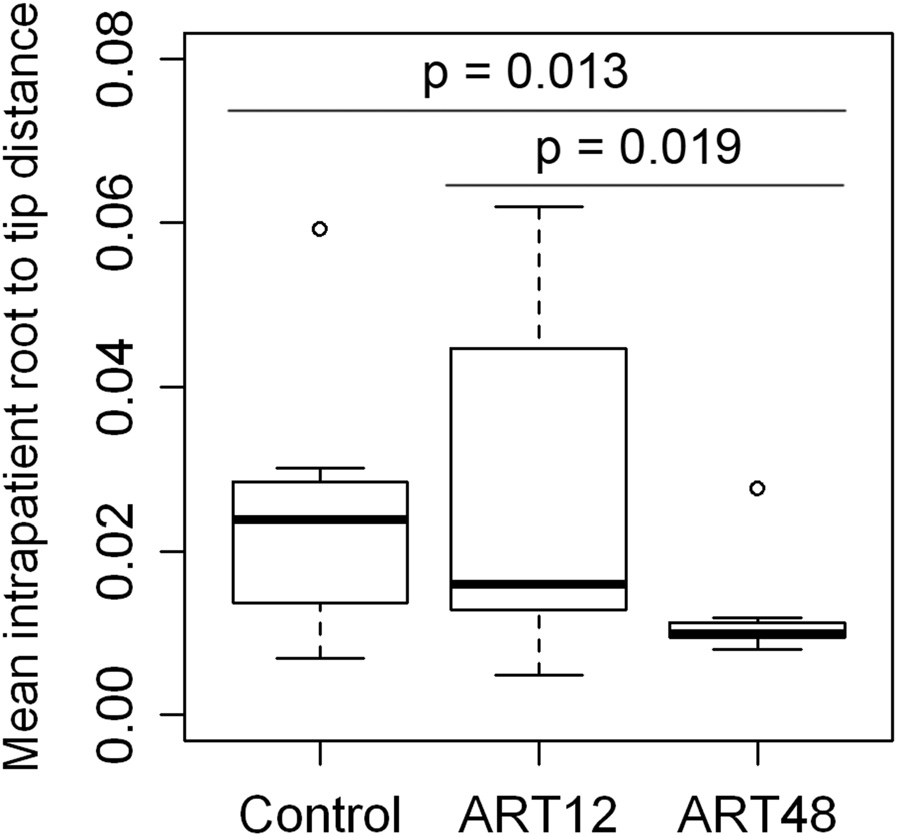
**Influence of ART on the divergence of the viral population.** Each group contains 10 individuals. Control, non-treated individuals; ART12, short course antiretroviral therapy (12 weeks); ART48, long course antiretroviral therapy (48 weeks). The divergence is shown as mean intra-patient root to tip distance, defined as genetic distance (in number of nucleotide substitutions per site) from each sequence to the root. Distances were calculated under the uncorrected (p) model of nucleotide substitution (number of substitutions accumulated along the path from the tip to the root, divided by the length of the alignment). Root to tip distances were analysed over a time of ~ 60 weeks after primary infection. The significance of differences in the mean between groups was tested by Wilcoxon rank sum tests. There is no significant difference between the control group and ART12 (*p* = 0.780).

The trial SPARTAC was designed to assess the clinical impact of short course (12 weeks, ART12) and long course (48 weeks, ART48) antiretroviral therapy initiated soon after seroconversion. To analyse the effects of ART12 and ART48 on the divergence of the viral population, (i.e. to assess how far the population has evolved from the founder virus), mean root to tip distances for sequences from each patient over a time of ~ 60 weeks were analysed and compared between the arms of the study (Figure 7). This time point corresponds to 12 weeks after the end of ART in the ART48 group, and 48 weeks after the end of ART in the ART12 group. Viral loads at ~ 60 weeks after primary infection had rebounded close to viral loads at primary infection in all subjects, with no significant differences in viral load between the non-treated control group and ART12 (*p* = 0.248), the control group and ART48 (*p* = 0.211) or ART12 and ART48 (*p* = 0.497) (Additional file 5: Figure S4). The divergence after ART48 was significantly lower compared to ART12 (*p* = 0.019) or the non-treated control group (*p* = 0.013), while no significant differences were found between ART12 and the control group (*p* = 0.780).

## Discussion

Deep sequencing using second generation sequencing technology [11] offers unprecedented possibilities to rapidly generate large and quantitative data and to detect minor variants of pathogens. Elucidation of the error rates of our procedures shows they are consistent with previous studies [13] with per-nucleotide error rates of 0.11% and 0.10%, respectively, for the new GS FLX Titanium chemistry. We determined that under our conditions recombinant sequences were observed less frequently, 3/11491 (0.03%) than the 0.11% and 0.15% found by Tsibris *et al*. [8]. Understanding the error associated with the entire sequencing process allowed the reliable analysis of sequence variants of the *env* V3 in a cross-sectional study of 30 patients at primary HIV infection. Our per-nucleotide error rate allowed the derivation of a frequency of detection for the least abundant sequence above experimental errors. This was defined as a frequency of 1.5%, and therefore all minor variants with frequencies above this cut-off were considered to be true variants.

The degree of sequence diversity of the *env* V3 was considerable among patients, with the proportion represented by the major variant ranging from 93.3% and 16.2% of the sequenced population. Although amplification was performed using primers in conserved regions of *env*, we cannot exclude the possibility of cis-linkage between the primer binding sites and the V3 region biasing our measures of diversity by preferentially amplifying one or more variants. Nevertheless our data are consistent with HTA assays which also revealed strong ‘monoclonal’ *env* bands in a number of individuals during early HIV infection [6,7]. Coreceptor usage prediction revealed that more than ¼ of the *env* V3 variants in subject 18 at primary infection were predicted as capable of using the CXCR4 coreceptor. Deep *env* V3 sequencing is therefore a valuable tool for the sensitive and quantitative assessment of HIV diversity and divergence as well as coreceptor tropism at primary infection [3].

Intra-individual differences in HIV population diversity are apparent at primary infection and here we arbitrarily assigned individuals into 3 diversity groups (low, medium and high) based on Shannon Entropy. The degree of HIV sequence diversity has been associated with CD4 counts later in infection [6]. Here, however, we were unable to correlate viral load, time from seroconversion, or CD4 counts with the frequency of sequence variants or the Shannon entropy of each individual’s HIV-1 *env* V3 population. As the patients included in this study were selected based on their detectable viral loads (average 223,737 RNA copies per ml at baseline), 1 ml plasma was used for RNA extraction and 10 μl RNA were used for the initial one-step RT-PCR, it is unlikely that a bias in sequence diversity was due to undersampling of the virus population during sample processing. However, it is clear that a 1 ml plasma sample from an adult represents only a fraction of the total blood volume of ~ 5 liters. In addition, focusing on post seroconversion samples may be too short a time frame to see longer term correlations with these other measures of HIV replication. Limiting the analysis to the *env* V3 may affect the power to find correlations with clinical markers of HIV infection.

Serial sampling of three individuals with high, medium and low diversity following primary infection suggests that increases in diversity and divergence from the virus present immediately following primary infection occurred as previously described [5], and these properties are detectable even over 60 weeks of sampling. The depth of sequencing obtained in our study allowed a much greater insight into the HIV population dynamics of these three individuals than it could be achieved by clonal sequencing. Subject 26, classed as low initial sequence diversity showed minimal increase in HIV divergence over time. Interestingly, at 419 days the re-emergence of variants occurred that were identical or closely related to variants present immediately post seroconversion. If this represents re-emergence from an archived provirus pool or the emergence of a variant continuously replicating in a different reservoir but excluded from the peripheral blood until day 419 is not clear. Re-emergence of archived variants has been documented following interruption from prolonged HAART [19,20], and deep sequencing of provirus populations from macrophages indicates higher and distinct HIV diversity than T lymphocytes [12]. It is likely therefore that combined deep sequence analysis of viral and proviral populations at primary infection will reveal more details of the intra-individual HIV evolution over time. Subject 17, classified with initial medium sequence diversity had an uncomplicated pattern of increasing HIV sequence divergence, and the emergence of different diverse populations over time, in agreement with previous analysis [5]. Subject 25, classed as high initial sequence diversity showed evidence of two distinct HIV populations at primary infection. Both virus lineages persisted following primary infection suggesting they are representative of viruses with equivalent fitness. Whether they represent the result of transmission of more than a single virus as has been observed in approximately 25% of primary infection [1], or are the result of a super-infection during seroconversion cannot be determined by deep sequencing of the *env* V3. Furthermore, no conclusions can be made about the other 27 subjects, as only two samples were analysed for each of these subjects.

As HIV sequence divergence is affected by immune response and antiviral therapy, we determined if differences in divergence were observable at ~ 60 weeks after primary infection in individuals on the short (12 weeks, ART12) or long (48 weeks, ART48) course antiviral therapy following seroconversion. The divergence of the population from the founder virus was significantly lower after ART48 than in the non-treated control group, consistent with the successful suppression of HIV-1 evolution during HAART as previously reported [9,10]. In contrast, ART12 did not have a significant effect on the divergence at ~ 60 weeks after primary infection. As viral loads are not significantly different on both treatment arms at ~ 60 weeks after primary infection, the observed difference in divergence between ART12 and ART48 most likely reflects prolonged virus replication inhibition in the ART48 arm. Therefore, virus divergence at ~ 60 weeks after primary infection provides a measure of the previous extent of antiretroviral suppression of HIV replication. These observed differences are unlikely to remain with continued virus replication in the absence of antiretroviral therapy. Whether more short lived effects on the ART12 arm are visible at earlier times is not clear, however the HIV evolutionary properties analysed here suggest a lack of efficacy of ART12 in the prolonged alteration of HIV evolutionary dynamics. Whether ART12 affects clinical markers of HIV infection and progression in the framework of the SPARTAC trial is currently under investigation.

## Conclusions

We conclude, after analysis of 80 patient samples and appropriate controls, that deep sequencing of the HIV-1 *env* V3 can successfully be applied to ascertain HIV-1 diversity in clinical settings. It shows detailed and complex intra-patient dynamics of *env* V3 diversity and divergence following primary infection. Importantly, we found that long course antiretroviral therapy for 48 weeks (ART48), initiated soon after seroconversion, clearly produces a measurable difference in HIV sequence evolution. Such therapy may therefore have a more pronounced impact on markers of HIV infection and progression than short course antiretroviral therapy (ART12). Our analysis of the *env* V3 as a marker for HIV-1 genome diversity is, therefore, informative in detecting HIV-1 treatment effects.

## Methods

### Subjects

Thirty study subjects diagnosed with primary HIV-1 infection (PHI) were selected from the Short Pulse Antiretroviral Therapy at Seroconversion (SPARTAC) study. PHI was confirmed by one of the following criteria: i) positive HIV-1 serology following a negative test within 6 months, ii) low level “incident” result using a detuned HIV-1 ELISA, iii) rising antibody levels detected by progression of protein bands on Western blot, or iv) positive HIV-1 proviral PCR or p24-antigen ELISA in the absence of HIV-1 specific antibodies [21]. The participants studied longitudinally were randomised three ways, to no therapy (Control) or 12 weeks (ART12) or 48 weeks (ART48) antiretroviral therapy shortly after seroconversion, with 10 subjects in each group. Only subjects with plasma viral loads greater than 30,000 RNA copies per ml at enrolment were selected for study. Samples collected at enrolment from subjects E6 and H8, who were not included in deep sequencing studies, were used to construct cloned HIV-1 envelopes for use as sequencing controls.

### Viral RNA extraction

Viral RNA was extracted from EDTA plasma collected at enrolment and after ~ 60 weeks from all subjects, and longitudinally over ~ 60 weeks from three subjects, using a QIAamp Viral RNA Mini Kit (Qiagen, Crawley, UK). Briefly, 1 ml of EDTA treated plasma was centrifuged for 1 hour at 25,000 g. The resultant pellet was resuspended in 140 μl nuclease-free phosphate buffered saline before continuing with the RNA purification according to the manufacturer’s instructions. RNA was eluted in 50 μl of elution buffer.

### *In vitro* transcription

Full length HIV-1 *env* was reverse transcribed and PCR amplified from plasma viral RNA from subjects E6 and H8 using primers EnvF (5^′^-GGG CTC GAG GCG GCC GCG AGC AGA AGA CAG TGG CAA TG-3^′^) and EnvR (5^′^-AAA TCT AGA GGG CCC CCA CTT GCC ACC CAT RTT A-3^′^). Amplified *env* genes were cloned into pcDNA3.1 (+) using restriction sites ApaI and XbaI. Single clones were purified from transformed MACH I strain E.coli using a GeneJET™ Plasmid Miniprep Kit (Fermentas, York, UK). Eight micrograms of each plasmid were linearised with 50 units of ApaI restriction enzyme and digested DNA was purified using a GeneJET™ Gel Extraction Kit (Fermentas). One-hundred nanograms of purified DNA were transcribed with T7 polymerase (Invitrogen, Paisley, UK) in the supplied buffer supplemented with 0.4 mM of each nucleotide, 100 ng/μl BSA (New England Biolabs, Hitchen, UK), 2 units/μl of RNaseOUT™ Ribonuclease Inhibitor (Invitrogen) and in the presence of 5 mM DTT. Reactions were incubated for 2 hours at 37°C. Template DNA was digested with one unit of DNAase I (Invitrogen) at 37°C for 30 min and reactions were terminated with a final concentration of 2.5 mM EDTA (pH 8.0) for 10 min at 65°C. Transcripts were purified using an RNeasy kit (Qiagen) with on-column DNase digestion according to the manufacturer’s instructions. The integrity and quantity of RNA was determined using a Bioanalyzer 2100 (Agilent Technologies, Wokingham, UK). Elimination of DNA template was confirmed by quantitative real-time PCR using SYBR Green PCR master mix (Sigma Aldrich, Gillingham, UK), which did not produce a fluorescent signal after 35 amplification cycles in a LightCycler 2.0 (Roche Applied Science, Lewes, UK).

### Reverse transcription and nested polymerase chain reaction

Viral RNA and *in vitro* transcribed RNA were reverse transcribed and PCR amplified in a single-tube reaction using SuperScript® III One-Step RT-PCR System with Platinum® Taq High Fidelity (Invitrogen). Each 50 μl reaction contained 200 nM of primer CV1 (5^′^-TAA CAT GTG GAA AAA TAA CAT GGT-3^′^), 200 nM of primer CV4 (5^′^-AGA AAA ATT CTC CTC CAC AAT TAA A-3^′^) and 10 μl plasma RNA extract or 10^4^ copies of RNA transcript. Cycling conditions were 55°C for 30 min (reverse transcription), 94°C for 2 min (hot start), 35 cycles of 94°C for 30 s, 55°C for 30 s, 68°C for 30 seconds, and a final incubation at 68°C for 5 min. Second round PCR was performed using Phusion® Hot Start High-Fidelity DNA Polymerase (Finnzymes). Each 25 ul reaction contained 12.25 ul Nuclease-Free Water, 5 ul Phusion® HF Buffer (5x), 0.5 ul dNTP mix (10 mM each), 1 ul each of primers JAE1 (5^′^-CAC AGT ACA ATG TAC ACA TG-3^′^) and JAE4 (5^′^-ACA ATT TCT GGG TCC CCT CC-3^′^) (20 pmol/ul), 0.25 ul Phusion® Hot Start DNA Polymerase (2 U/μl) and 5 ul of template first round PCR product. Cycling conditions were 98°C for 30 s (initial denaturation), 40 cycles of 98°C for 10 s, 60°C for 20 s, 72°C for 15 s, and a final incubation at 72°C for 10 min. Amplicons with a size of 387 bp were visualized by 2% agarose gel electrophoresis for 1 h at 120 V in Tris-acetate-EDTA buffer.

### Deep sequencing of amplicons

Nested PCR amplicons were sequenced without further purification using the Genome Sequencer FLX Instrument and GS FLX Titanium series reagents (Roche/454 Life Sciences) according to the manufacturer’s instructions. In short, single-stranded DNA libraries were prepared with the GS FLX Titanium Rapid Library Preparation Kit, using one of the 12 Multiplex Identifier (MID) adaptors for each sample. Between 5 and 12 libraries were pooled and immobilized onto DNA capture beads. Clonal amplification was carried out by emulsion PCR. Emulsions were broken, beads were enriched, loaded onto 1/4 or 1/8 of a PicoTiterPlate and sequencing-by-synthesis was performed [11].

### Read assembly and filtering

One SFF file per region of the PicoTiterPlate was generated by the Genome Sequencer FLX System Software (Roche/454 Life Sciences). Using the sfffile and sffinfo commands of the SFF tools, the SFF file was split into separate SFF files based on the MIDs, and FASTA files containing sequence reads for each sample were created. Sequence reads were mapped against a HIV-1 subtype B *env* sequence which matched the samples with regard to the country of origin (UK) and the collection date (2005) [GenBank: FJ653428] using the software Segminator [18,22]. Thereby reads with a length < 70 nt and/or low identity to the reference sequence were removed. The pipeline for data processing, filtering and assembly is summarised in Additional file 6: Figure S5. Alignments were trimmed to the *env* V3 region and visually inspected in BioEdit version 7.0.9.0 [23]. All reads not spanning the entire *env* V3 region as well as misaligned reads were removed. To obtain a final alignment, reads containing ‘Ns‘were removed, as these correspond to positions where no nucleotide was incorporated during sequencing in greater than three consecutive flows. Additional file 1: Table S1 summarizes how many sequences were discarded at each step of the filtering process. Subsequently, duplicate sequences were eliminated with the ElimDupes tool [24] which generated an alignment comprising only the unique sequences found in each sample, and a table showing the numbers and names of the duplicates represented by each unique sequence. Frequencies of sequence variants as well as process error rates for the control samples were calculated and the most frequent unique sequences within each sample were extracted from the alignments with a combination of Excel and customized python™ scripts. All figures were computed in R version 2.10.1 [25].

### Recombination analysis

The Highlighter tool [26] was used to visualize alignments and to screen for recombinant sequences. These were verified with the manual bootscan method [27] implemented in the Recombination Detection program version 3.42 [28,29].

### Shannon information entropy

Sequence diversity was assessed by measuring the Shannon information entropy [30] and calculated with a customized R script. Shannon Entropy at time t is defined as

(1) $$S(t)=-\sum_{i=1}^{N(t)}f_{i}(t)log_{2}\;f_{i}(t)$$

where *N(t)* is the number of unique sequence variants that are present at time *t*, and *f*_*i*_*(t)* is the proportion of the *i*^th^ sequence variant [4]. The more diverse the variants present in amounts comparable to one another, the greater the resulting entropy value.

### Genetic distance and divergence analysis and Neighbor-joining trees

To analyse the dynamics of sequence divergence of the *env* V3 following primary infection in three patients in detail, sequence divergence was calculated as the pairwise genetic distance (number of nucleotide substitutions per site) of a sequence variant at a given time from the consensus sequence at time 0. Prior to computation, the appropriate model of nucleotide substitution was determined using jModelTest version 0.1.1 [31]. Genetic distances were estimated under the General Time Reversible model of nucleotide substitution, with proportion of invariable sites and gamma-distributed rate heterogeneity (GTR + I + G), using PAUP* version 4.0b10 [32] and Neighbor-joining trees were reconstructed under the same model. Trees were edited with the program FigTree version 1.3.1 [33].

To explore the impact of ART on divergence of the viral population over time in all 30 patients, mean intra-patient root to tip distances, defined as genetic distance (in number of nucleotide substitutions per site) from each sequence to the root, were used. Distances were calculated under the uncorrected (p) model of nucleotide substitution using PAUP* version 4.0b10. The null hypothesis that the root to tip distances are equal in the three groups was tested using Wilcoxon rank sum tests using R version 2.10.1.

### Coreceptor usage prediction

Coreceptor usage of sequence variants was predicted using both position-specific scoring matrices (PSSM) [34] and support vector machines (SVM) implemented in the Geno2pheno [coreceptor] tool [35].

### Accession numbers for sequencing data

The Roche/454 Life Sciences sequencing data obtained in this study is available from the EMBL/GenBank®/DDBJ Sequence Read Archive under study accession number ERP001147.

## Competing interests

The authors declare that they have no competing interests.

## Authors’ contributions

SJF, JNW, MOM, PK conceived the study. AG, SK, DB, RR performed the experiments. AG, SH, GJB, SJF analysed the data. AG, SK, PK wrote the paper. All authors read and approved the final manuscript.

## SPARTAC Investigators

### Trial Steering Committee (TSC)

*Independent TSC Members:* A Breckenridge (Chair), P Clayden, C Conlon, F Conradie, J Kaldor*, F Maggiolo; F Ssali

*Country Principal Investigators:* D A Cooper, P Kaleebu, G Ramjee, M Schechter, G Tambussi, J Weber

### Trial Physician

Sarah Fidler

### Trial Statistician

Abdel Babiker

### Data and Safety Monitoring Committee (DSMC)

T Peto (Chair) A McLaren (in memoriam), V Beral, G Chene, J Hakim

### Co-ordinating Trial Centre

MRC Clinical Trials Unit, London (A Babiker, K Porter, M Thomason, F Ewings, M Gabriel, D Johnson, K Thompson, A Cursley*, K Donegan*, E Fossey*, P Kelleher*, K Lee*, B Murphy*, D Nock*)

### Central Immunology Laboratories and Repositories

The Peter Medawar Building for Pathogen Research, University of Oxford, UK (R Phillips, J Frater, L Ohm Laursen*, N Robinson, P Goulder, H Brown)

### Central Virology Laboratories and Repositories

Jefferiss Trust Laboratories, Imperial College, London, UK (M McClure, D Bonsall*, O Erlwein*, A Helander*, S Kaye, M Robinson, Lisa Cook*, Gemma Adcock*, Parvin Ahmed*)

### Clinical Endpoint Review Committee

N Paton, S Fidler

### Investigators and Staff at Participating Sites

*Australia:* St Vincent’s Hospital, Sydney (A Kelleher), Northside Clinic, Melbourne (R Moore), East Sydney Doctors, Sydney, (R McFarlane), Prahran Market Clinic, Melbourne (N Roth), Taylor Square Private Clinic, Sydney (R Finlayson), The Centre Clinic, Melbourne (B Kiem Tee), Sexual Health Centre, Melbourne (T Read), AIDS Medical Unit, Brisbane (M Kelly), Burwood Rd Practice, Sydney (N.Doong) Holdsworth House Medical Practice, Sydney (M Bloch) Aids Research Initiative, Sydney (C Workman).

*Coordinating centre in Australia*: Kirby Institute University of New South Wales, Sydney (P Grey, D A Cooper, A Kelleher, M Law).

*Brazil*: Projeto Praça Onze, Hospital Escola São Francisco de Assis, Universidade federal do Rio de Janeiro, Rio de Janeiro (M Schechter, P Gama, M Mercon*, M Barbosa de Souza, C Beppu Yoshida, J R Grangeiro da Silva, A Sampaio Amaral, D Fernandes de Aguiar, M de Fátima Melo, R Quaresma Garrido

*Italy:* Ospedale San Raffaele, Milan (G Tambussi, S Nozza, M Pogliaghi, S ChiappettaL Della Torre, Elisa Gasparotto,), Ospedale Lazzaro Spallanzani, Roma (G D’Offizi, C Vlassi, A Corpolongo)

South Africa:

*Cape Town*: Desmond Tutu HIV Centre*,* Institute of Infectious Diseases, Cape Town

(R Wood, J Pitt, C Orrell, F Cilliers, R Croxford, K Middelkoop, L G Bekker, C Heiberg, J Aploon, N Killa, E Fielder, T Buhler)

*Johannesburg*: The Wits Reproductive Health and HIV Institute, University of Witswatersrand, Hillbrow Health Precinct, Johannesburg. (H Rees, F Venter, T Palanee) Contract Laboratory Services, Johannesburg Hospital, Johannesburg (W Stevens, C Ingram, M Majam, M Papathanasopoulos)

*Kwazulu-Natal*: HIV Prevention Unit, Medical Research Council, Durban (G Ramjee, S Gappoo, J Moodley, A Premrajh, L Zako)

*Uganda:* MRC/Uganda Virus Research Institute, Entebbe (H Grosskurth, A Kamali, P Kaleebu, U Bahemuka, J Mugisha*, H F Njaj*)

*Spain:* Hospital Clinic-IDIBAPS. University of Barcelona, Barcelona (J M Miro, M López-Dieguez*, C. Manzardo, JA Arnaiz, T. Pumarola, M. Plana, M. Tuset, MC Ligero, MT García, T. Gallart, JM Gatell)

*UK and Ireland:* Royal Sussex County Hospital, Brighton (M Fisher, K Hobbs, N Perry, D Pao, D Maitland, L Heald), St James’s Hospital, Dublin (F Mulcahy, G Courtney, S O’Dea, D Reidy), Regional Infectious Diseases Unit, Western General Hospital and Genitourinary Dept, Royal Infirmary of Edinburgh, Edinburgh (C Leen, G Scott, L Ellis, S Morris, P Simmonds), Chelsea and Westminster Hospital, London (B Gazzard, D Hawkins, C Higgs), Homerton Hospital, London (J Anderson, S Mguni), Mortimer Market Centre, London (I Williams, N De Esteban, P Pellegrino, A Arenas-Pinto, D Cornforth*, J Turner*) North Middlesex Hospital (J Ainsworth, A Waters), Royal Free Hospital (M Johnson, S Kinloch, A Carroll, P Byrne, Z Cuthbertson), Barts & the London NHS Trust, London (C Orkin, J Hand, C De Souza), St Mary’s Hospital, London (J Weber, S Fidler, E Hamlyn, E Thomson*, J Fox*, K Legg, S Mullaney*, A Winston, S Wilson, P Ambrose), Birmingham Heartlands Hospital (S Taylor, G Gilleran).

### Imperial College Trial Secretariat

S Keeling, A Becker

### Imperial College DSMC Secretariat

C Boocock

Left the study team before the trial ended

## Supplementary Material

Additional file 1: Table S1 Read numbers in data analysis steps. Click here for file

Additional file 2: Figure S1 Phred quality scores of non-consensus bases in the *env* V3 region. Data from 80 patient samples and 3 control samples were analysed by determining the median, interquartile range and maximum/minimum Phred scores for each nucleotide in each sequence with each boxplot representing one sample. No boxplots are shown for 3 patient samples and 2 control samples, as no minor variants above the 1.5% frequency cut-off were found in these. The median Phred quality score was 32.50 with a range from 21.17 to 39.86. Click here for file

Additional file 3: Figure S2 Coreceptor usage prediction for the *env* V3 variants in patient and control samples. Predictions were made for the 12 most frequent sequence variants found in each of the 30 patients as well as the two control samples by (a) the PSSM method applying the X4R5 matrix and (b) the SINSI matrix or (c) the Geno2pheno [coreceptor] tool. The colour of the fields in (a) and (b) represents the determined PSSM scores ranging from association with CCR5 usage (red) or CXCR4 usage (white) or in (c) the false positive rate of the prediction of CXCR4 usage. The black line stands for the cut-off defined in this study: based on analysis of the control samples, minor variants with frequencies of > 1.5% are considered to be true. *, sequence contains a stop codon. Click here for file

Additional file 4: Figure S3 Correlation of the Shannon Entropy with the number of sequence variants. The correlation is shown for 30 subjects at primary infection with HIV. Patients were classified into three groups based on the sequence diversity. Group 1 is defined by a low sequence diversity (Shannon Entropy 0–0.75) and is shown as stars. Group 2 is defined by a medium sequence diversity (Shannon Entropy 0.75–1.5) and is shown as triangles. Group 3 is defined by a high sequence diversity (Shannon Entropy > 1.5) and is shown as dots. Click here for file

Additional file 5: Figure S4 Viral load at ~ 60 weeks after primary infection with HIV. Each group contains 10 individuals. Control, non-treated individuals; ART12, short course antiretroviral therapy (12 weeks); ART48, long course antiretroviral therapy (48 weeks). The significance of differences in the mean between groups was tested by Wilcoxon rank sum tests. There is no significant difference between the non-treated control group and ART12 (*p* = 0.248), the control group and ART48 (*p* = 0.211) or ART12 and ART48 (*p* = 0.497). Click here for file

Additional file 6: Figure S5 Data processing, filtering and assembly. Workflow for data analysis of 454 short read amplicons. Click here for file
