# Supplementary material for: Restriction of V3 region sequence divergence in the HIV-1 envelope gene during antiretroviral treatment in a cohort of recent seroconverters
Source: Retrovirology. 2013 Jan 18;10:8. doi: 10.1186/1742-4690-10-8 (PMC3605130; doi:10.1186/1742-4690-10-8)
Supplement: Additional file 1: Table S1 — Read numbers in data analysis steps. [file 1742-4690-10-8-S1.pdf]

Table S1 Read numbers in data analysis steps

Sample	Initial no. of reads	Included in assembly[a]		Comprising complete V3[b]		Final alignment[c]		Unique sequences	
		No.	% of total reads	No.	% of total reads	No.	% of total reads	No.	% of final alignment
**Subject (time) - samples at primary infection**									
**#1 (day 0)**	1629	1531	93.98	1197	73.48	1093	67.10	114	10.43
**#2 (day 0)**	13214	10900	82.49	8505	64.36	8351	63.20	902	10.80
**#3 (day 0)**	9361	7583	81.01	6130	65.48	6098	65.14	338	5.54
**#4 (day 0)**	12109	8818	72.82	7054	58.25	6891	56.91	385	5.59
**#5 (day 0)**	7621	6463	84.81	4925	64.62	4894	64.22	255	5.21
**#6 (day 0)**	8564	5878	68.64	4598	53.69	4545	53.07	410	9.02
**#7 (day 0)**	8327	6609	79.37	5615	67.43	5540	66.53	301	5.43
**#8 (day 0)**	1015	970	95.57	806	79.41	793	78.13	56	7.06
**#9 (day 0)**	1410	1283	90.99	1187	84.18	1185	84.04	100	8.44
**#10 (day 0)**	2705	2573	95.12	2365	87.43	2355	87.06	83	3.52
**#11 (day 0)**	3772	3358	89.02	2979	78.98	2967	78.66	154	5.19
**#12 (day 0)**	3932	2783	70.78	1306	33.21	1279	32.53	95	7.43
**#13 (day 0)**	2293	2227	97.12	2008	87.57	2001	87.27	119	5.95
**#14 (day 0)**	1996	1801	90.23	1410	70.64	1370	68.64	171	12.48
**#15 (day 0)**	508	462	90.94	405	79.72	403	79.33	23	5.71
**#16 (day 0)**	9822	7936	80.80	5542	56.42	5175	52.69	299	5.78
**#17 (day 0)**	37555	24341	64.81	16709	44.49	16394	43.65	712	4.34
**#18 (day 0)**	12650	11362	89.82	8381	66.25	8026	63.45	652	8.12
**#19 (day 0)**	17282	6492	37.57	3928	22.73	3845	22.25	440	11.44
**#20 (day 0)**	24562	11566	47.09	8558	34.84	8498	34.60	395	4.65
**#21 (day 0)**	28233	11136	39.44	6975	24.71	6865	24.32	309	4.50
**#22 (day 0)**	2592	2319	89.47	1267	48.88	1211	46.72	128	10.57
**#23 (day 0)**	2351	1597	67.93	1094	46.53	1077	45.81	165	15.32

**#24 (day 0)**	2654	1609	60.63	1425	53.69	1411	53.17	159	11.27
**#25 (day 0)**	12069	11259	93.29	6504	53.89	6369	52.77	796	12.50
**#26 (day 0)**	2196	1987	90.48	1395	63.52	1328	60.47	116	8.73
**#27 (day 0)**	4869	4439	91.17	2412	49.54	2354	48.35	157	6.67
**#28 (day 0)**	3600	3145	87.36	2148	59.67	2073	57.58	161	7.77
**#29 (day 0)**	1905	1573	82.57	1116	58.58	1108	58.16	152	13.72
**#30 (day 0)**	1959	1910	97.50	1215	62.02	1214	61.97	51	4.20
**Subject (time) - samples at week 60**									
**#1 (day 436)**	3776	3715	98.38	3322	87.98	3275	86.73	83	2.53
**#2 (day 437)**	5453	5263	96.52	4023	73.78	4015	73.63	437	10.88
**#3 (day 441)**	1963	1825	92.97	1425	72.59	1424	72.54	100	7.02
**#4 (day 435)**	2440	2335	95.70	2060	84.43	2058	84.34	94	4.57
**#5 (day 438)**	2689	2553	94.94	2062	76.68	2055	76.42	105	5.11
**#6 (day 422)**	1882	1732	92.03	1600	85.02	1597	84.86	99	6.20
**#7 (day 450)**	4243	4065	95.80	3653	86.09	3639	85.76	253	6.95
**#8 (day 445)**	16818	11818	70.27	9480	56.37	9270	55.12	733	7.91
**#9 (day 419)**	19742	17558	88.94	14841	75.17	14810	75.02	300	2.03
**#10 (day 429)**	5239	4839	92.36	4289	81.87	4284	81.77	126	2.94
**#11 (day 434)**	10447	7169	68.62	6762	64.73	6582	63.00	309	4.69
**#12 (day 449)**	15818	13614	86.07	11599	73.33	11545	72.99	306	2.65
**#13 (day 420)**	3460	3217	92.98	2508	72.49	2446	70.69	266	10.87
**#14 (day 413)**	12525	11607	92.67	9019	72.01	8698	69.45	662	7.61
**#15 (day 431)**	1628	1323	81.27	1073	65.91	1037	63.70	59	5.69
**#16 (day 433)**	2473	2316	93.65	1748	70.68	1618	65.43	226	13.97
**#17 (day 420)**	6864	6120	89.16	5156	75.12	5000	72.84	658	13.16
**#18 (day 392)**	1268	1211	95.50	925	72.95	882	69.56	161	18.25
**#19 (day 415)**	3030	2391	78.91	1793	59.17	1776	58.61	181	10.19

**#20 (day 468)**	15952	11282	70.72	8934	56.01	8872	55.62	410	4.62
**#21 (day 443)**	30536	23431	76.73	17316	56.71	16753	54.86	795	4.75
**#22 (day 442)**	3646	2995	82.14	2159	59.22	2013	55.21	211	10.48
**#23 (day 387)**	22652	20761	91.65	16169	71.38	15706	69.34	787	5.01
**#24 (day 438)**	2811	676	24.05	479	17.04	476	16.93	62	13.03
**#25 (day 421)**	9852	5166	52.44	1041	10.57	1026	10.41	314	30.60
**#26 (day 419)**	7602	5225	68.73	1192	15.68	1183	15.56	167	14.12
**#27 (day 421)**	6332	3479	54.94	1450	22.90	1432	22.62	190	13.27
**#28 (day 429)**	3633	1957	53.87	910	25.05	904	24.88	181	20.02
**#29 (day 440)**	7387	4490	60.78	3597	48.69	3546	48.00	462	13.03
**#30 (day 427)**	16825	14116	83.90	11178	66.44	10887	64.71	504	4.63
**Subject (time) - serial samples between primary infection and week 60**									
**#26 (day 8)**	27346	25675	93.89	23655	86.50	23302	85.21	356	1.53
**#26 (day 34)**	27373	25676	93.80	23268	85.00	22792	83.26	422	1.85
**#26 (day 63)**	19527	18063	92.50	16629	85.16	16244	83.19	312	1.92
**#26 (day 90)**	18753	17035	90.84	7448	39.72	6450	34.39	323	5.01
**#26 (day 219)**	39681	36466	91.90	16711	42.11	14180	35.73	523	3.69
**#26 (day 255)**	18273	16948	92.75	6788	37.15	5809	31.79	330	5.68
**#26 (day 318)**	41616	35032	84.18	15544	37.35	13704	32.93	531	3.87
**#26 (day 328)**	22577	19805	87.72	8618	38.17	7555	33.46	401	5.31
**# 17 (day 49)**	23556	21623	91.79	11658	49.49	11079	47.03	822	7.42
**# 17 (day 84)**	9149	8262	90.30	4251	46.46	4060	44.38	411	10.12
**# 17 (day 119)**	14202	12627	88.91	5716	40.25	5481	38.59	527	9.62
**# 17 (day 175)**	12851	11069	86.13	4706	36.62	4513	35.12	635	14.07
**# 17 (day 266)**	19425	17213	88.61	7210	37.12	6764	34.82	807	11.93
**# 17 (day 357)**	33844	29222	86.34	16464	48.65	15519	45.85	1471	9.48
**# 25 (day 2)**	2149	1013	47.14	412	19.17	354	16.47	112	31.64

**# 25 (day 35)**	56360	30955	54.92	26035	46.19	25854	45.87	2074	8.02
**# 25 (day 98)**	22306	20125	90.22	18285	81.97	18162	81.42	724	3.99
**# 25 (day 175)**	58021	34467	59.40	29546	50.92	29323	50.54	2374	8.10
**# 25 (day 287)**	88144	31801	36.08	26822	30.43	26604	30.18	2313	8.69
**# 25 (day 367)**	67407	34082	50.56	29595	43.90	29387	43.60	2087	7.10
**Mean**	13929	10141	80.22	7129	57.91	6908	56.40	437	8.42
**Control samples**									
**E6**	15908	11222	70.54	9826	61.77	9783	61.50	213	2.18
**H8**	19215	18040	93.88	15602	81.20	15524	80.79	279	1.80
**E6:H8**	15739	11763	74.74	11515	73.16	11491	73.01	297	2.58
**Mean**	16954	13675	79.72	12314	72.04	12266	71.77	263	2.19

[a] Short reads and reads with low identity to reference sequence were removed. A maximum of 40000 reads was used.

[b] Assemblies were visually inspected and misaligned reads removed.

[c] Reads containing "Ns" were removed.
