# Supplementary figures and images for: Restriction of V3 region sequence divergence in the HIV-1 envelope gene during antiretroviral treatment in a cohort of recent seroconverters
Source: Retrovirology. 2013 Jan 18;10:8. doi: 10.1186/1742-4690-10-8 (PMC3605130; doi:10.1186/1742-4690-10-8)

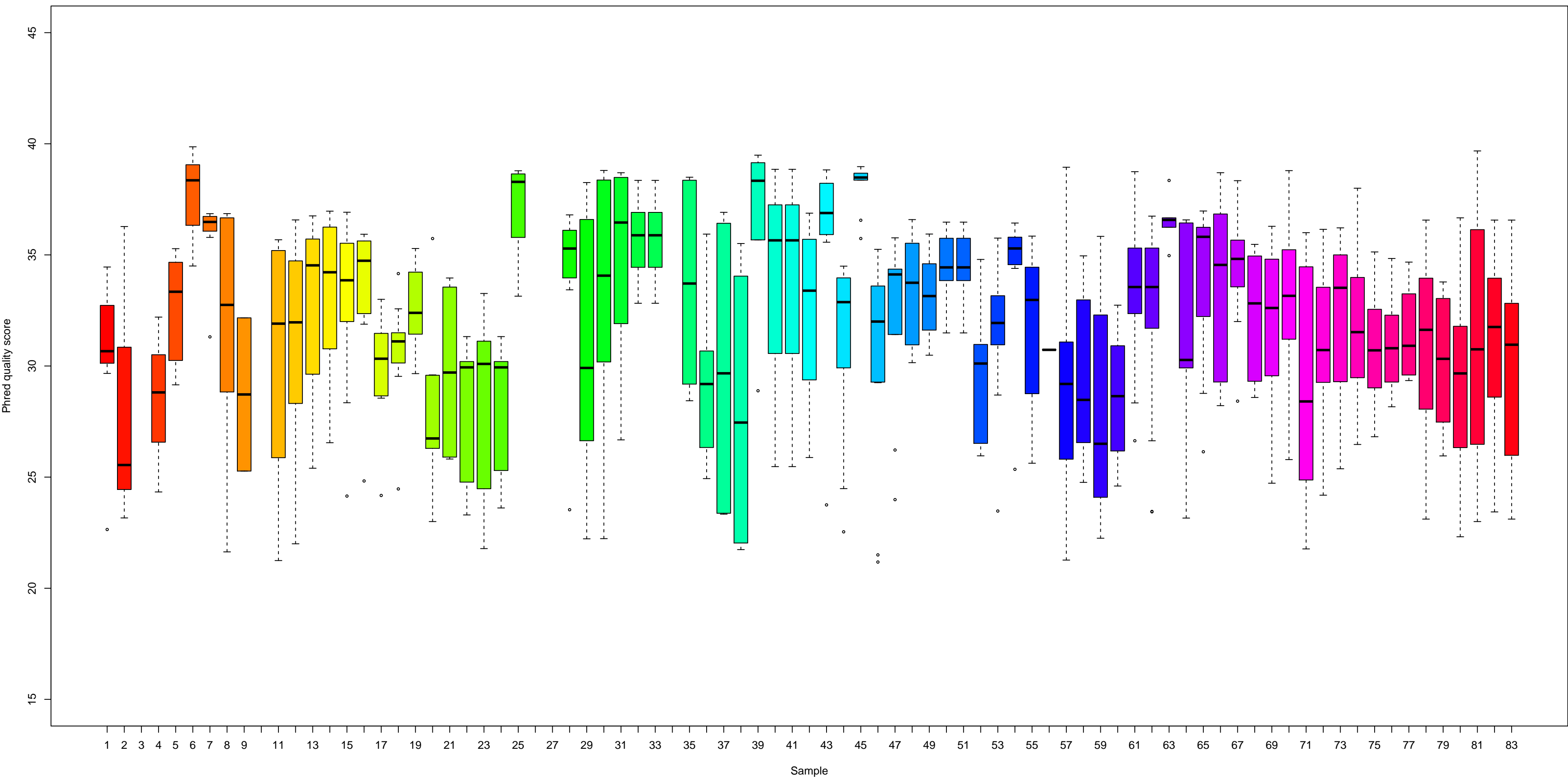

Supplement: Additional file 2: Figure S1 — Phred quality scores of non-consensus bases in the env V3 region. Data from 80 patient samples and 3 control samples were analysed by determining the median, interquartile range and maximum/minimum Phred scores for each nucleotide in each sequence with each boxplot representing one sample. No boxplots are shown for 3 patient samples and 2 control samples, as no minor variants above the 1.5% frequency cut-off were found in these. The median Phred quality score was 32.50 with a range from 21.17 to 39.86. [file 1742-4690-10-8-S2.pdf]

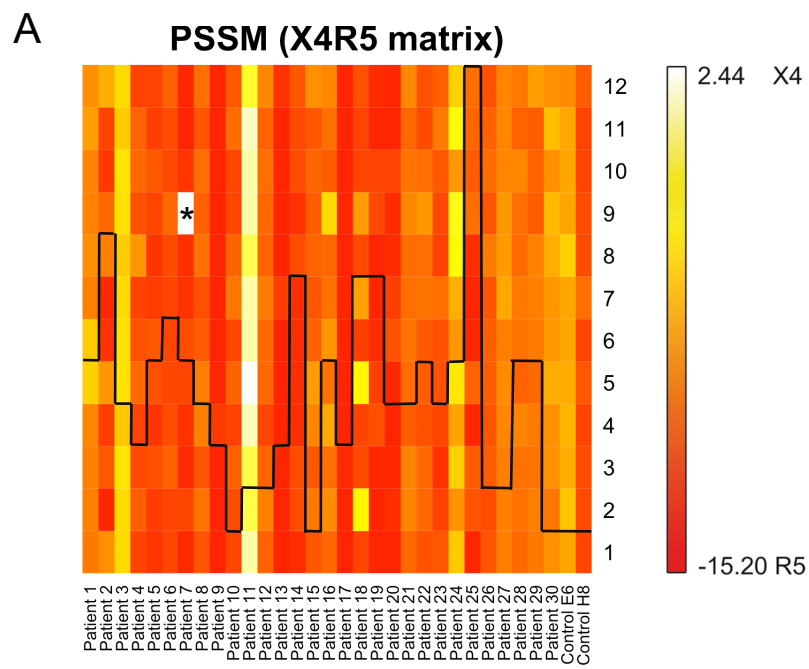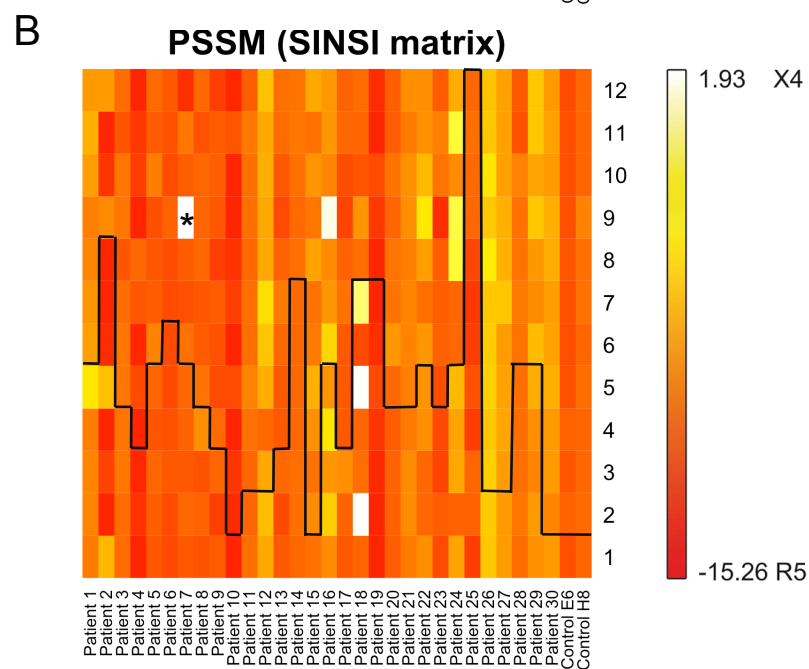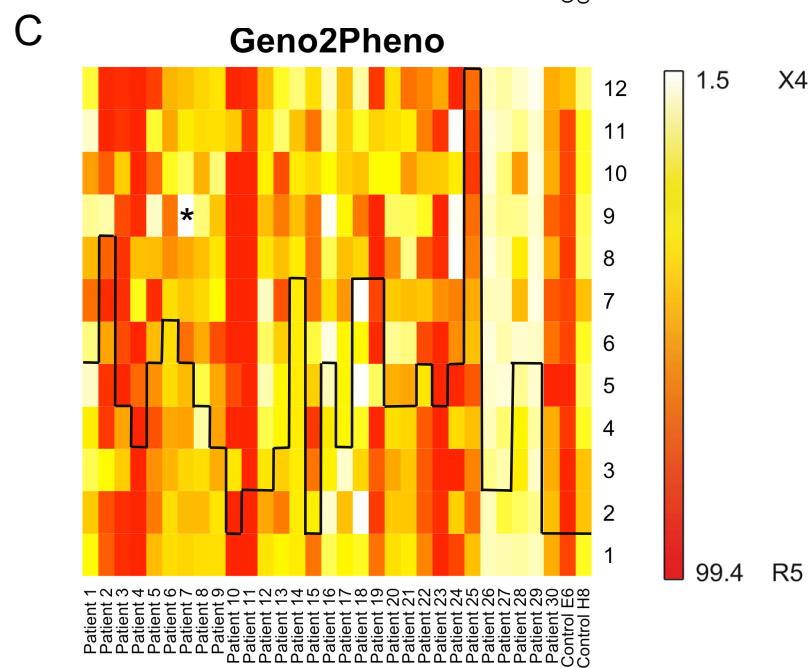

Supplement: Additional file 3: Figure S2 — Coreceptor usage prediction for the env V3 variants in patient and control samples. Predictions were made for the 12 most frequent sequence variants found in each of the 30 patients as well as the two control samples by (a) the PSSM method applying the X4R5 matrix and (b) the SINSI matrix or (c) the Geno2pheno [coreceptor] tool. The colour of the fields in (a) and (b) represents the determined PSSM scores ranging from association with CCR5 usage (red) or CXCR4 usage (white) or in (c) the false positive rate of the prediction of CXCR4 usage. The black line stands for the cut-off defined in this study: based on analysis of the control samples, minor variants with frequencies of > 1.5% are considered to be true. *, sequence contains a stop codon. [file 1742-4690-10-8-S3.pdf]

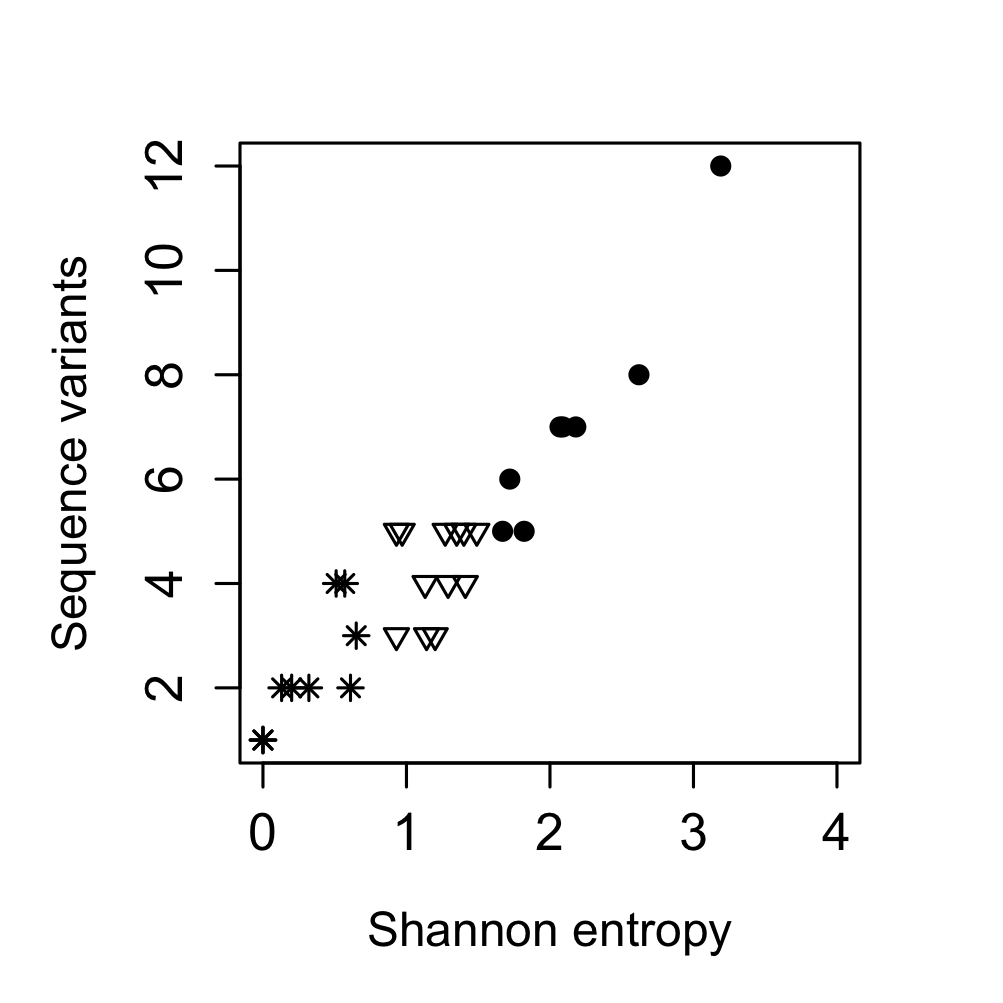

Supplement: Additional file 4: Figure S3 — Correlation of the Shannon Entropy with the number of sequence variants. The correlation is shown for 30 subjects at primary infection with HIV. Patients were classified into three groups based on the sequence diversity. Group 1 is defined by a low sequence diversity (Shannon Entropy 0–0.75) and is shown as stars. Group 2 is defined by a medium sequence diversity (Shannon Entropy 0.75–1.5) and is shown as triangles. Group 3 is defined by a high sequence diversity (Shannon Entropy > 1.5) and is shown as dots. [file 1742-4690-10-8-S4.tiff]

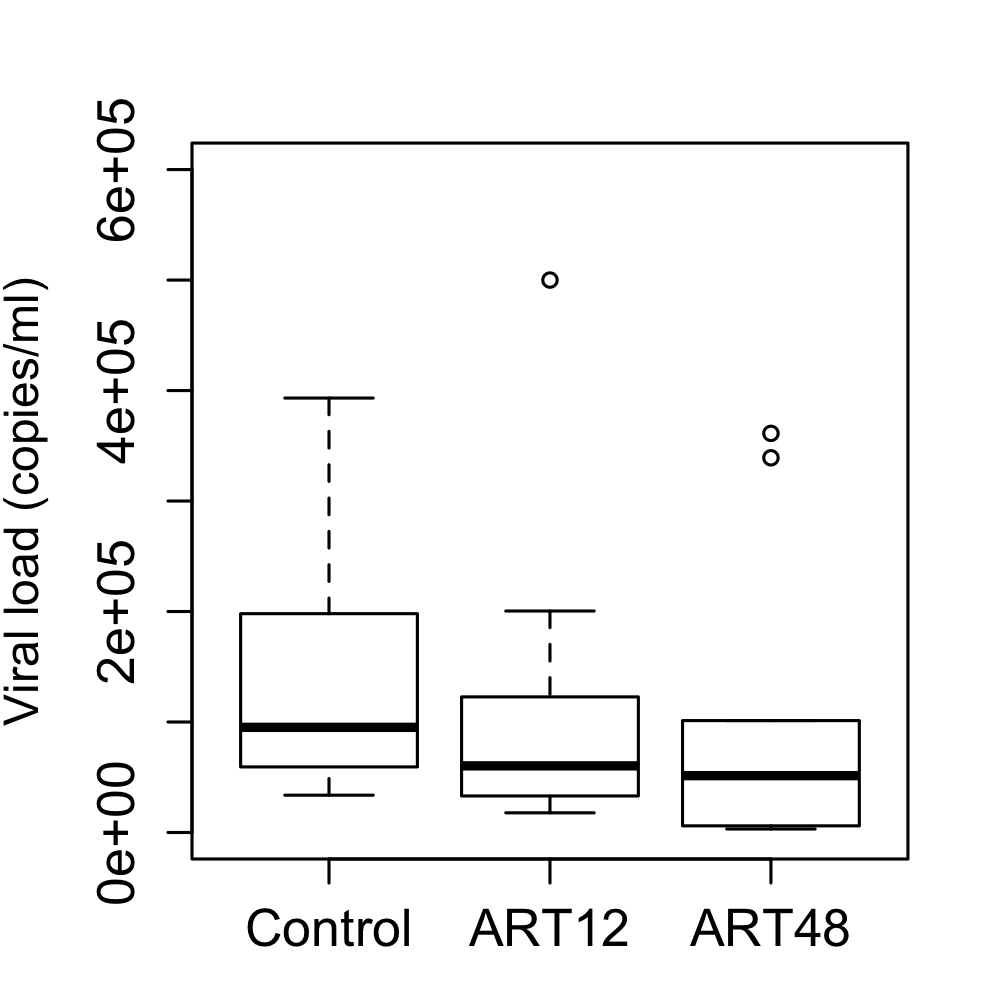

Supplement: Additional file 5: Figure S4 — Viral load at ~ 60 weeks after primary infection with HIV. Each group contains 10 individuals. Control, non-treated individuals; ART12, short course antiretroviral therapy (12 weeks); ART48, long course antiretroviral therapy (48 weeks). The significance of differences in the mean between groups was tested by Wilcoxon rank sum tests. There is no significant difference between the non-treated control group and ART12 (p = 0.248), the control group and ART48 (p = 0.211) or ART12 and ART48 (p = 0.497). [file 1742-4690-10-8-S5.tiff]
