# Supplementary material for: Restriction of V3 region sequence divergence in the HIV-1 envelope gene during antiretroviral treatment in a cohort of recent seroconverters
Source: Retrovirology. 2013 Jan 18;10:8. doi: 10.1186/1742-4690-10-8 (PMC3605130; doi:10.1186/1742-4690-10-8)
Supplement: Additional file 6: Figure S5 — Data processing, filtering and assembly. Workflow for data analysis of 454 short read amplicons. [file 1742-4690-10-8-S6.pdf]

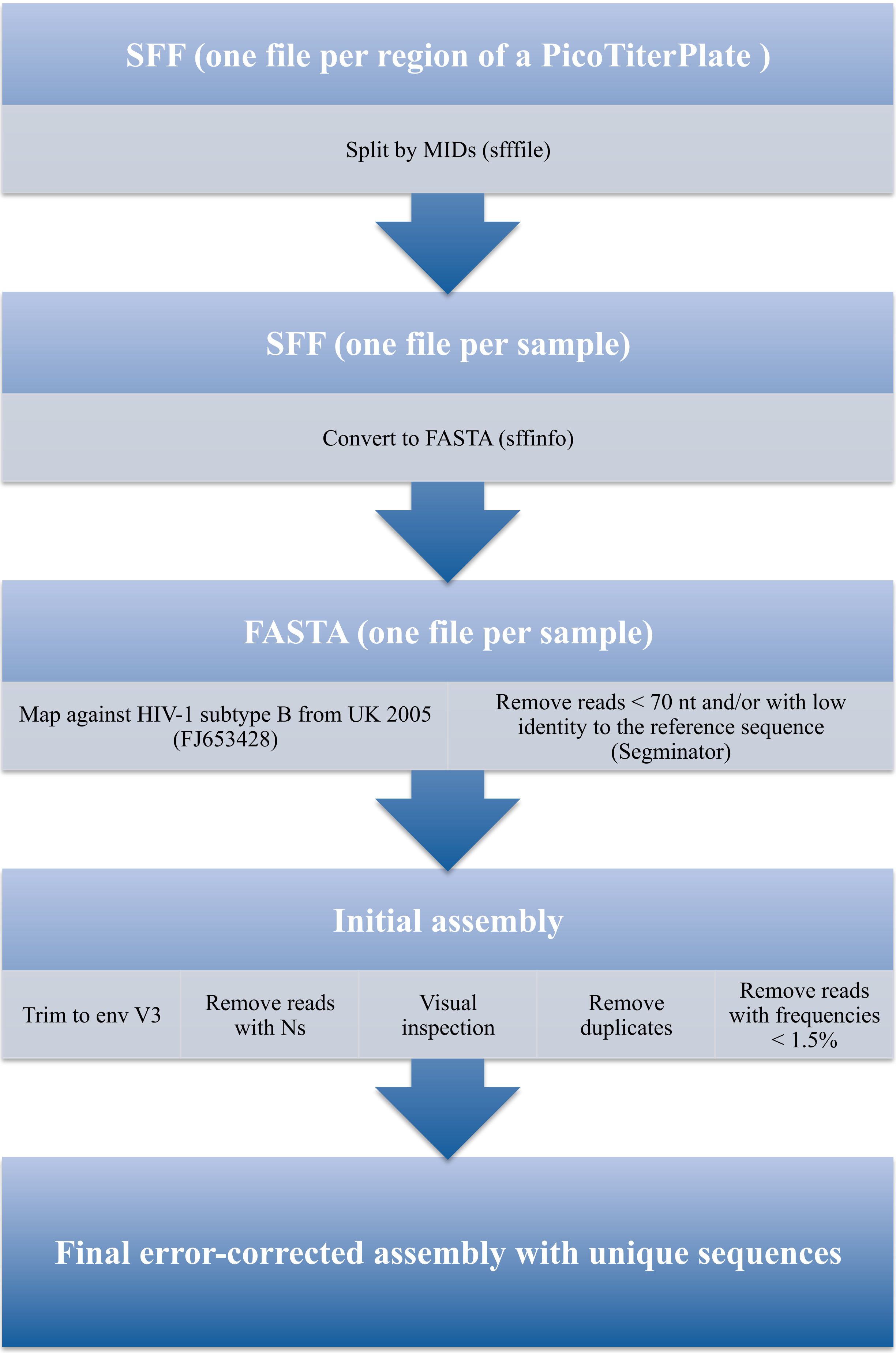
SFF (one file per region of a PicoTiterPlate)
Split by MIDs (sfffile)
SFF (one file per sample)
Convert to FASTA (sffinfo)
FASTA (one file per sample)
Map against HIV-1 subtype B from UK 2005 (FJ653428)
Remove reads < 70 nt and/or with low identity to the reference sequence (Segminator)
Initial assembly
Trim to env V3
Remove reads with Ns
Visual inspection
Remove duplicates
Remove reads with frequencies < 1.5%
Final error-corrected assembly with unique sequences
